# Effects of Dimethylaminoethanol and Compound Amino Acid on D-Galactose Induced Skin Aging Model of Rat

**DOI:** 10.1155/2014/507351

**Published:** 2014-07-14

**Authors:** Su Liu, Zhenyu Chen, Xia Cai, Ying Sun, Cailing Zhao, Fangjun Liu, Dalie Liu

**Affiliations:** ^1^Department of Plastic Surgery, Zhujiang Hospital, Southern Medical University, 253 GongYe Middle Avenue, Guangzhou, Guangdong 510280, China; ^2^Department of Plastic and Aesthetic Surgery, The Affiliated Hospital of Medical School, Qingdao University, Qingdao, Shandong 266003, China; ^3^Department of Dermatology, Huang-Si Aesthetic Surgery Hospital, Beijing 100120, China; ^4^Center for Advanced Orthopedic Studies, Beth Israel Deaconess Medical Center, Harvard Medical School, Boston, MA 02215, USA; ^5^Institute of Plastic and Reconstructive Surgery, Weifang Medical University, Shandong 261042, China

## Abstract

A lasting dream of human beings is to reverse or postpone aging. In this study, dimethylaminoethanol (DMAE) and compound amino acid (AA) in Mesotherapy were investigated for their potential antiaging effects on D-galactose induced aging skin. At 18 days after D-gal induction, each rat was treated with intradermal microinjection of saline, AA, 0.1% DMAE, 0.2% DMAE, 0.1% DMAE + AA, or 0.2% DMAE + AA, respectively. At 42 days after treatment, the skin wound was harvested and assayed. Measurement of epidermal and dermal thickness in 0.1% DMAE + AA and 0.2% DMAE + AA groups appeared significantly thicker than aging control rats. No differences were found in tissue water content among groups. Hydroxyproline in 0.1% DMAE + AA, 0.2% DMAE + AA, and sham control groups was much higher than all other groups. Collagen type I, type III, and MMP-1 expression was highly upregulated in both 0.1% DMAE + AA and 0.2% DMAE + AA groups compared with aging control. In contrast, TIMP-1 expression levels of various aging groups were significantly reduced when compared to sham control. Coinjection of DMAE and AA into target tissue has marked antiaging effects on D-galactose induced skin aging model of rat.

## 1. Introduction

Antiaging is an eternal topic and dream of human being. Age-related skin changes are inevitable and include thinning, sagging, wrinkling, loss of elasticity, areas of dryness, and an inversed turnover of collagen type I/III ratio in the skin which presented as reduced synthesis of collagen type I but upregulated production of collagen type III [1]. Currently Mesotherapy has been arousing everyone's interest as an antiaging strategy. It is a minimally invasive procedure, which consists of intradermal microinjection of pharmacologic substances, such as nutrients, hormones, vitamins, enzymes, and other reagents, that have been diluted and are administered directly into the region to be treated. Under sterile and professional manipulation, Mesotherapy is very rarely causing troubles of skin infection and necrosis, except some minor risks like swelling and pain during the injection. As a safe, simple, less painful procedure which is one of the so-called “lunchtime cosmetic procedures,” it requires no recovery time and is perfect for professionals and successful people in the fast-paced modern life [2–4].

Dimethylaminoethanol (DMAE), an analog of the B vitamin choline and a precursor of acetylcholine, has been receiving more attention as an exciting new skincare supplement today for its acute effects of antiaging, antiwrinkle, and skin firmness. DMAE is well known for use in external application. In the randomized clinical studies, 3% DMAE facial gel has been shown to be safe and efficacious in the mitigation of forehead lines and periorbital fine wrinkles, and in improving lip fullness and shape and the overall appearance of facial skin [5, 6]. An open-label extension of the trial also showed that the long-term application of DMAE gel for up to 1 year was associated with a good safety profile [7]. However, tropical treatment with DMAE usually requires high dose and concentration to pass through epidermal permeability barrier, which could incur concerns of its toxicity, side effects, and medical costs. It was reported that 2.5–10 mmol/mL DMAE could cause a vacuolar cytopathology of in vitro cultured human fibroblast cells. In addition, studies showed that application of 3% DMAE gel tropically could also incur the vacuolar cytopathology of rabbit ear epidermal cells. Alternative delivery of DMAE is needed to evaluate the relative efficacy for the improvement of aging skin.

In order to evaluate potential antiaging effects of low-dose DMAE administered intradermally by localized microinjection (Mesotherapy), tissue structure and collagen metabolism of D-gal induced aging skin were measured in this study. Meanwhile, coinjection of compound amino acid (AA), leading to a reduced cellular toxicity by DMAE injection and to be a local nutrition supply, was studied as well. Their considered mechanism of action in the skin was also described.

## 2. Materials and Methods

### 2.1. Animals

Male Wistar rats were purchased from Institute of pharmaceutical Sciences (Qingdao, China). All animals weighted 180–220 g at the time of surgery. Animal care and experimental protocol were approved by the Affiliated Hospital of Qingdao University Medical School Institutional Animal Care and Use Committee.

Total 80 Wistar rats were randomly divided into each experimental group (as detailed in Table 1). Animals from each treatment group, except sham control group, were given subcutaneous injection with D-galactose (D-gal) at the dose of 125 mg/kg*·*d for 6 weeks to induce the skin aging model of rat. At 18 days after D-gal injection, each rat was anesthetized with chloral hydrate (0.25 mL per 100 g body weight) and disinfected within its hip area. A 3 cm round tattoo area was prefabricated on each side of rat's hip and then treated under general anesthesia once a week for 4 weeks with intradermal microinjection of saline (NS), AA, 0.1% DMAE, 0.2% DMAE, 0.1% DMAE + AA, or 0.2% DMAE + AA, respectively. Both aging negative control and sham control groups received exactly the same surgical procedure but without any treatment under study. At 42 days after treatment, all animals were euthanized and skin wounds were compared by histology, measurement of water content, hydroxyproline content, and quantitative real-time PCR testing.

### 2.2. Histopathology

Skin thickness was measured with SimplePCI Image analysis software (Hamamatsu Corporation, Boston, MA) in sections stained with Hematoxylin and eosin. Epidermal thickness was measured from the stratum corneum (SC) surface to the bottom of the dermal papillae, while dermal thickness was measured from the top of the papillae to the deepest portions of the reticular dermis until adipose tissue or muscle was reached. Under the same magnification, 5 individual positions were randomly selected to measure the skin thickness from each of 5 fields of view per section. With Masson Trichrome stain of all sections, 5 different fields of view were also randomly chosen and analyzed for collagen fiber density.

### 2.3. Measurement of Water Content

1 cm^2^ skin wound was harvested and measured for the precise wet weigh, followed by baking at 80°C for 12 hours before measuring its dry weight. Skin water content was calculated by the formula of the percentage of water content = ((wet weight − dry weight) ÷ wet weight) × 100%.

### 2.4. Measurement of Hydroxyproline Content

Hydroxyproline content in skin wound was measured according to the manufacture's protocol of commercial assay kits (NANJING JIANCHEN biological engineering Co., LTD, Nanjing, China).

### 2.5. Quantitative Reverse Transcription-Polymerase Chain Reaction (qRT-PCR)

Skin wounds were homogenized in TRIzol1 (Invitrogen) and total RNA was extracted according to manufacturer's protocol. 1 *μ*g total RNA was reversely transcribed into cDNA using the PrimeScript RT Synthesis System (Takara Biotechnology/Takara Bio, Dalian, China). A highly sensitive quantitative PCR method was performed with these cDNA products. All customized primers and probes were validated for their efficiency (as shown in Table 2). The 10 mL reaction mix contained 2x Power SYBR1 Green PCR Master Mix, 10-fold diluted cDNA, and 0.2 mM of each primer. The thermal protocol consisted of 10 min polymerase activation at 95°C, followed by 40 cycles of denaturation at 95°C for 30 s, primer annealing at 55°C for 1 min, and extension at 72°C for 30 s. The mRNA expression levels were normalized to those of the endogenous reference gene *β*-action and presented as fold changes to those obtained from aging control skin samples.

### 2.6. Statistical Analysis

Data were expressed as means ± standard deviation (SD). Statistical analysis was performed by one-way analysis of variance (ANOVA) using SPSS17.0 software. The results were taken to be statistically significant at a probability level of *P* < 0.05.

## 3. Results

### 3.1. Histological Changes

**Figure 1:**
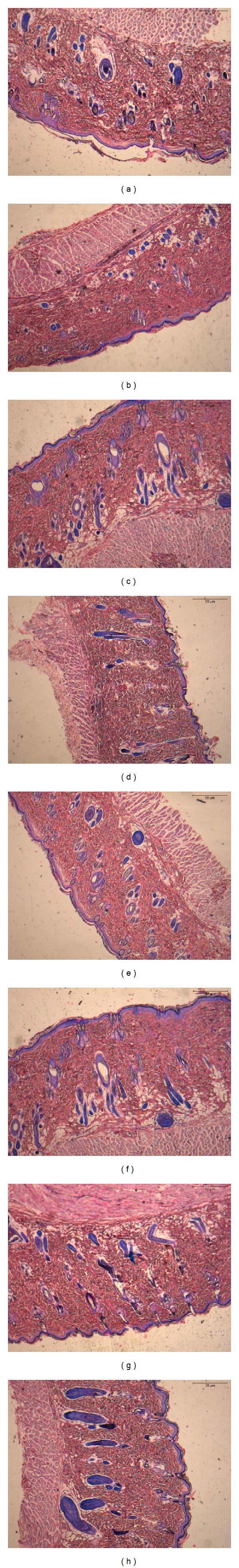
Histological appearance of skin wound from D-gal induced aging model of rats. Skin wounds were harvested for histology at 42 days after each treatment. Representative images of sections stained with H&E were presented at a low magnification (40x, scale bar = 50 *μ*m). Letters (a) through (h) correspond to aging control, aging NS, aging AA, aging 0.1% DMAE, aging 0.2% DMAE, aging 0.1% DMAE + A, aging 0.2% DMAE + AA, and sham control, respectively.

Aging skin showed decreased thickness for both epidermis and dermis when compared to sham control rats. With aging, the epidermis reduced its numbers of cell layers, and the dermal collagen fibers turned to be sparse, slender, or broken. When compared to aging control, however, these histologic changes were greatly ameliorated in 0.1% DMAE + AA, 0.2% DMAE + AA, and 0.2% DMAE groups (Figures 1(a)–1(h)).

**Figure 2:**
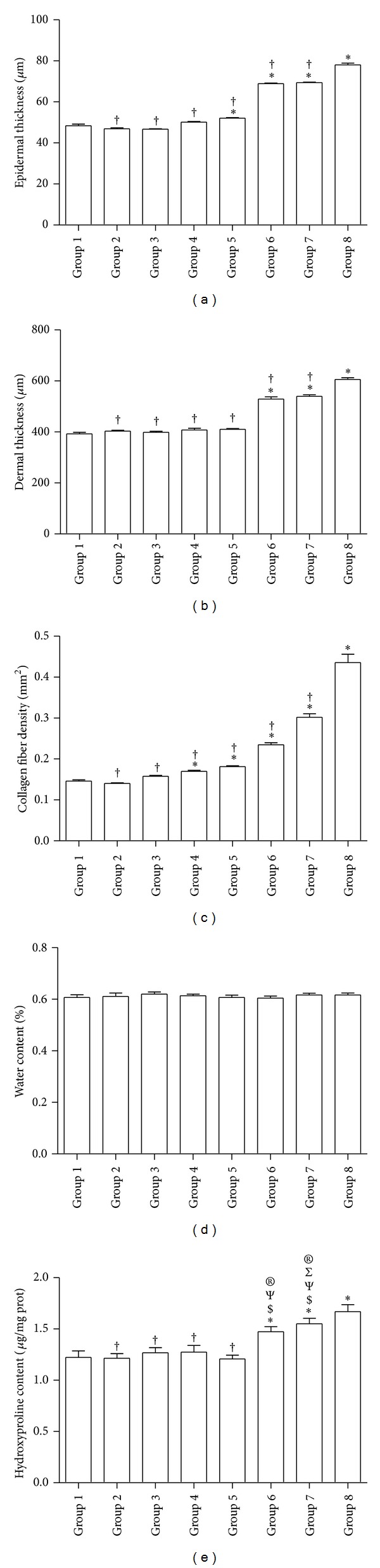
Physical properties of D-gal induced aging skin wound after each treatment. (a) Epidermal thickness (*μ*m), (b) dermal thickness (*μ*m), (c) collagen fiber density (mm^2^), (d) water content (%), (e) hydroxyproline content (*μ*g/mg protein). ∗, †, $, Ψ, Σ, and ® denote a significant difference (*P* < 0.05) relative to negative control, sham control, NS, AA, 0.1% DMAE, and 0.2% DMAE, respectively. For water content assay, no significant difference existed among all treatment groups.

When treated with 0.1% DMAE + AA or 0.2% DMAE + AA, both epidermal and dermal thickness and density of collagen fiber were significantly elevated over those of aging control group. In addition, 0.2% DMAE alone also significantly increased epidermal thickness and density of collagen fiber when compared to aging control. But, nevertheless, these three parameters in all aging groups were greatly downregulated when compared to those of sham control group (Figures 2(a), 2(b), and 2(c)).

### 3.2. Water Content in Aging Skin

There were no statistically significant differences in water content among all groups (Figure 2(d)).

### 3.3. Hydroxyproline Content in Aging Skin

Hydroxyproline content were greatly increased in aging skin treated with both 0.1% DMAE + AA and 0.2% DMAE + AA complex, which showed significant differences when compared to all other aging groups. Interestingly, the hydroxyproline content in 0.2% DMAE + AA group reached an equivalent level to that of sham control but was significantly higher than all other 6 aging groups. There was no significant difference between 0.1% DMAE + AA and 0.2% DMAE + AA groups (Figure 2(e)).

### 3.4. mRNA Expression of Type I, Type III Procollagen, MMP-1, and TIMP-1 Target Genes

**Figure 3:**
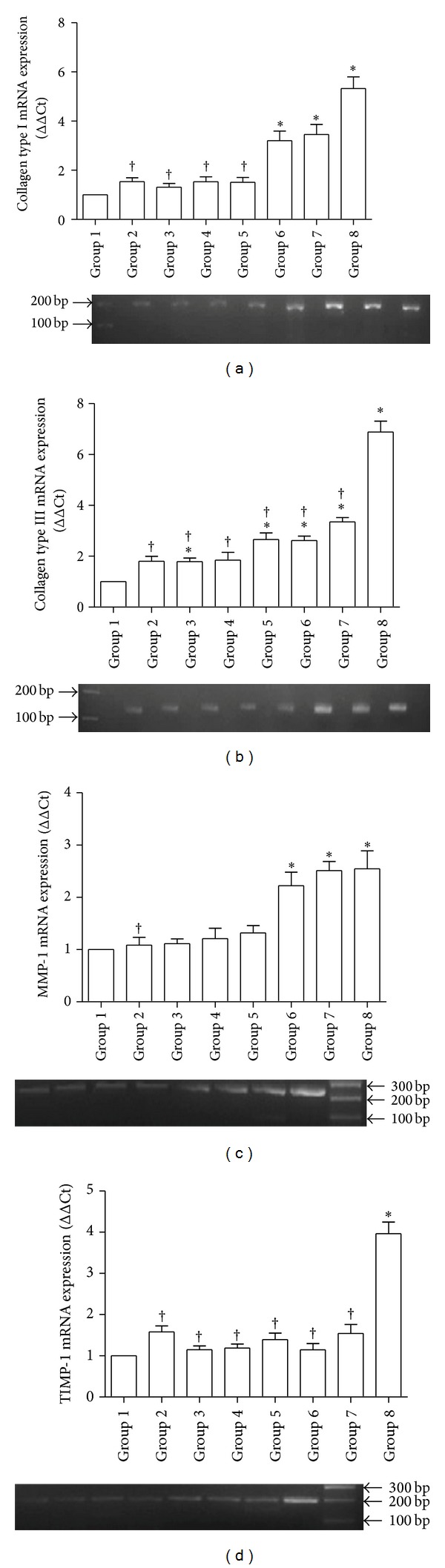
Measurement for collagen metabolism of D-gal induced aging skin after each treatment. Skin wound was processed and analyzed for expression of collagen type I (a), collagen type III (b), MMP-1 (c) and its inhibitor, and TIMP-1 (d) by quantitative RT-PCR. Expression levels for each targeting gene were normalized to endogenous reference gene and reported as relative values (ΔΔCt) to those obtained from aging control group. ∗ and † indicate a significant difference (*P* < 0.05) when compared to negative control (group 1) and sham control (group 8), respectively.

Collagen type I expression was greatly upregulated in response to both 0.1% DMAE + AA and 0.2% DMAE + AA treatments, which showed no significant difference to that of sham control. However, any of the other aging groups showed much less expression of collagen type I when compared to sham control rats (Figure 3(a)). When treated with 0.1% DMAE + AA, 0.2% DMAE + AA, or 0.2% DMAE alone, transcript levels for collagen type III were considerably elevated over aging control group, but significantly less than sham control (Figure 3(b)). As a potential key regulator in skin aging, MMP-1 messages increased in response to either 0.1% DMAE + AA or 0.2% DMAE + AA treatment, which showed a statistically significant difference from that of aging control, but not sham control rats (Figure 3(c)). In contrast, transcript levels for TIMP-1 in all aging groups were significantly downregulated to a much lower level of that of sham control (Figure 3(d)).

## 4. Discussion

Injection of low-dose D-galactose into rat skin could induce changes that resemble accelerated aging. In our studies, the aging model of rat showed hair color changes, decreased activities, and neurological impairment. The histologic changes of D-gal induced aging skin presented as thinner epidermis with reduced cells layers and sparse, slender, or broken collagen fibers in dermal layer. Furthermore, this aging skin indicated lower levels of hydroxyproline content and mRNA messages for type I, type III collagen, MMP-1, and TIMP-1 compared to normal skin tissue. All these data suggest that injection of 125 mg/kg*·*d D-galactose for 6 weeks is technically feasible to create a subacute aging model for mimicking human aging skin [8, 9].

Our studies indicated that coinjection of DMAE and AA increased the levels of hydroxyproline content and collagen type I expression of D-gal induced aging skin, which reached approximately the same level of normal skin tissue when given high dose of 0.2% DMAE + AA treatment. The histological changes appeared as elevated epidermal and dermal thickness and tightly arranged dermal collagen fibers. More interestingly, Mesotherapy by coinjection of DMAE and AA greatly elevated MMP-1 gene expression in aging skin to an equivalent level of normal rats, but not for its inhibitor of TIMP-1 that was significantly downregulated in all aging rats. These results suggest that Mesotherapy by coinjection of DMAE and AA played a positive regulation on collagen catabolism in the repair and reconstruction of aging skin and delay the aging process of skin. Previous studies showed the reduced production of collagen type I and increased synthesis of collagen type III and MMP-1 in aging skin [10]; however, our results indicated that mRNA expression for collagen type III and MMP-1 was much less in aging control than that of normal rats. It might be that collagen metabolism undergoes a different way between normal skin changes with aging and D-gal-induced subacute skin aging model. Our study also showed that injection of DMAE or AA alone showed no effects on hydroxyproline content or messages for type I collagen and MMP-1 in aging skin. These data indicate that the concomitant use of DMAE and AA might be the only way to exert their antiaging action in this D-gal induced aging skin model, by modulating collagen type I metabolism and remolding the structure of aging skin.

Previous studies have shown that cultured rabbit dermal fibroblasts responded to DMAE by massive vacuolization in a concentration-dependent manner. The epidermis of rabbit external ear was also significantly thickened and exhibited clear perinuclear swelling indicative of vacuolization in response to topical application of 3% DMAE. It was suggested that vacuolar cytopathology may not be dissociable from the improvement of skin appearance that is rapidly produced by topically administered DMAE and could be the cellular basis of the antiwrinkle effect of DMAE [11, 12]. From our results, the vacuolization of the dermal fibroblast might occur in both 0.1% and 0.2% DMAE treated aging skin, resulting in cellular swelling and increased collagen fiber density. Epithelial cells of aging skin could also respond to the higher concentration of 0.2% DMAE by vacuolization, which presented as increased epidermal thickness. However, these may need further studies to clarify this matter.

In addition, the measurement of skin water content showed no differences among all treated rats, which suggests that DMAE and compound AA had limited effects on skin moisturizing. This might be due to a lower level of concentration and shorter action period of local available DMAE in epidermal layer when direct subcutaneous injection was used for DMAE delivery, which differs from topical application of DMAE.

## 5. Conclusions

With D-galactose induced aging model of rat, Mesotherapy by delivering DMAE and AA directly to target tissue has marked antiaging effects by promoting collagen synthesis and catabolism to remodel skin texture and improving the thickness of aging skin. Although these experiments used a rat model, the results are relevant to clinical works. Further investigations to understand the mechanism of the action of dimethylaminoethanol and compound amino acid in aging skin will assist in the development and test of more powerful and effective skincare regimens for further human evaluation.

## Figures and Tables

**Table 1:** Experiment design (NS: 0.9% sodium chloride; AA: 3.48% compound amino acid; DMAE: dimethylaminoethanol).

	Group number	Pretreatment (D-galactose, 6 weeks)	Microinjection (once a week, 4 weeks)	Size (*n*)
Aging groups	1	125 mg/kg*·*d	Negative control	10
	2	125 mg/kg*·*d	NS	10
	3	125 mg/kg*·*d	AA	10
	4	125 mg/kg*·*d	0.1% DMAE	10
	5	125 mg/kg*·*d	0.2% DMAE	10
	6	125 mg/kg*·*d	0.1% DMAE + AA	10
	7	125 mg/kg*·*d	0.2% DMAE + AA	10

Sham control	8			10

**Table 2:** Primer pairs for target and housekeeping genes for quantitative RT-PCR assay.

Target gene accession number	Sequences		Probe length
	Forward	Reverse	
Col *α*1 I *NM_053304.1*	ATGTCTGGTTTGGA GAGAGCA	GAGGAGCAGGGAC TTCTTGAG	203 bp
Col *α*1 III *NM_032085.1*	GCCTCCCAGAACAT TACATACC	TTTGCTATTTCCTTC AGCCTTG	132 bp
MMP-1*α* *NM_001134530.1*	CTCCCTTGGACTCA CTCATTCTA	AGAACATCACCTCT CCCCTAAAC	227 bp
TIMP-1 *NM_053819.1*	TGGCATCCTCTTGTT GCTATC	CGAATCCTTTGAGC ATCTTAGTC	191 bp
*β*-Action *NM_031144.3 *	GGAGATTACTGCCC TGGCTCCTA	GACTCATCGTACTC CTGCTTGCTG	150 bp
